# Breast milk to blood lead ratios among women from the West Bank of Palestine: a cross-sectional study of associated factors

**DOI:** 10.1186/s13006-021-00410-3

**Published:** 2021-08-23

**Authors:** Ramzi Shawahna

**Affiliations:** 1 Department of Physiology, Pharmacology and Toxicology, Faculty of Medicine and Health Sciences, An-Najah National University, Nablus, Palestine; 2 An-Najah BioSciences Unit, Centre for Poisons Control, Chemical and Biological Analyses, An-Najah National University, Nablus, Palestine

**Keywords:** Heavy metals, Environmental, Exposure, Blood, Milk, Palestine

## Abstract

**Background:**

Infants fed contaminated breast milk are at an increased risk of exposure to lead. Breast milk to blood (M/B) ratios have not been investigated among women in Palestine. The aim of this study was to assess blood, breast milk, and M/B lead ratios in samples collected from Palestinian breastfeeding women. Associations between sociodemographic characteristics with breast milk lead levels and M/B lead ratios were also investigated.

**Methods:**

This study was conducted in a cross-sectional design in the period between October 2017 and April 2018. Breastfeeding women visiting maternity care centers in different regions of the West Bank of Palestine were recruited to the study by the nurses in the maternity care centers. Sociodemographic characteristics, venous blood, and breast milk samples were collected from each participant. Lead concentrations were analyzed using a validated inductively coupled plasma-mass spectrometric method. Mann–Whitney *U* test, Pearson’s Chi-square, Fisher’s exact, and Spearman’s correlations were used to analyze the data. Odds ratios (OR) were computed using a multivariate logistic regression model.

**Results:**

Matching blood and milk samples were collected from 80 women. Lead concentrations in 11 (13.8%) of the breast milk samples were above the World Health Organization’s recommended levels. Breast milk lead levels were more likely to be ≥5 μg/L in breastfeeding women who lived in urban areas (aOR 4.96; 95% CI 1.10, 22.38) compared to those who lived in rural areas. Breast milk to blood lead ratios were more likely to be ≥25% in breastfeeding women who lived in urban areas (aOR 7.06; 95% CI 1.68, 29.77), used *eye kohl* (aOR 14.29; 95% CI 1.32, 155.06), and used hair dye (aOR 5.33; 95% CI 1.58, 18.00) compared to those who lived in rural areas, did not use *eye kohl*, and did not use hair dye, respectively.

**Conclusions:**

Higher M/B lead ratios were predicted by living in urban areas, using *eye kohl*, and using hair dye. Decision makers in health authorities should address sources of exposure to lead in urban areas. Cosmetics containing lead should be assessed and regulated for lead content.

**Supplementary Information:**

The online version contains supplementary material available at 10.1186/s13006-021-00410-3.

## Background

Human breast milk is the ideal form of enteral nutrition for all infants [1]. It is widely accepted that human breast milk can provide all nutritional elements needed for normal growth of infants. On the other hand, human milk can also be a transfer medium for many toxic and undesirable elements from breastfeeding mothers to their infants. Heavy metals are among these toxic and undesirable elements that can be transferred from breastfeeding mothers to their infants via breast milk [2]. Lead is one of the most widely distributed heavy metal in our environment. It is well-established that exposure to lead is a hazard that can have health-related deleterious consequences. Exposure to lead has no known biological benefits. On the contrary, it has been demonstrated that exposure to lead can be associated with neurotoxicity, immunotoxicity, hematotoxicity, and nephrotoxicity in humans [3, 4]. Although it is difficult to establish a safe level of lead in biological samples, international health organizations including the World Health Organization recommend that lead levels should be below 5 μg/dL and 5 μg/L in the blood and breast milk, respectively [5]. Therefore, the Agency for Toxic Substances and Disease Registry (ASTDR) has placed lead in the list of top 10 priority toxic materials [6]. As a result of increasing urbanization and industrialization around the globe, people are continuously exposed to lead at both environmental and occupational levels. Although children are more vulnerable to the health-related consequences of exposure to lead, it is noteworthy that exposure to lead can also have serious consequences in adults. Previous studies have shown that exposure to lead can be harmful to the organ systems, notably, the central nervous system [3, 4].

In the Middle East, battery factories, smelters, repair shops, traditional stone mills, and burning of wastes are the major sources of lead in the environment [7]. People can become exposed to lead in the environment once they have ingested or inhaled a source of lead. Lead can then distribute to various body tissues [4]. Studies have shown that lead can be stored in teeth, trabecular, and cortical bones [3, 4]. During pregnancy, lead is mobilized from its stores to the blood. Consequently, lead can cross the placenta and reach to the developing fetus. Studies have shown that lead can also be sequestered into breast milk [2]. Although children could have been exposed to lead in utero as a result of maternal exposure to an environmental or occupational source of lead, lead-contaminated breast milk can be an additional source of lead exposure to breastfed infants [7, 8]. Lead has the potential to interfere with the development of the blood brain barrier, thus can easily cross to the brain and have neurotoxic consequences [3, 4].

In countries with loose regulations about contaminants, lead is still widely used in unregulated industries. Previous studies have detected lead in paints, clay utensils, cosmetics, and other materials used in agriculture [9, 10]. Therefore, people are continuously at risk of exposure to lead. Previous studies have assessed lead levels in blood, plasma, breast milk, saliva, and other biological samples [7, 11–13]. A systematic review of studies conducted in Iran showed that the vast majority of breast milk samples contained lead levels above the World Health Organization’s recommended level (< 5 μg/L) [13]. In Palestine, about one in five breast milk samples collected in the time period between May and April 2015 contained lead above the World Health Organization’s recommended level [7]. Another study reported that industrial workers in Palestine were environmentally and occupationally exposed to lead as indicated by the salivary samples collected in the time period between December 2017 and March 2018 [12].

It has been suggested that transfer of lead from plasma to milk appeared to be high at even low plasma levels. However, other studies reported low (less than 3%) maternal plasma and bone to breast milk lead ratios, suggesting that the barrier of the mammary gland is efficient enough to hinder transfer of lead from maternal blood and bone to breast milk [2, 14]. It is noteworthy to mention that previous studies have reported significantly variable breast milk to blood (M/B) lead ratios [2, 14–16]. The heterogeneity in M/B lead ratios was attributed to differences in exposure among the populations studied and analytical methods used to quantify lead [16].

Although lead levels were assessed in breast milk samples from Palestinian women in a previous study [7], M/B lead ratios were not investigated. Moreover, little is known on the factors that could be associated with high M/B lead ratios. Therefore, this study was conducted to assess blood, breast milk, and M/B lead ratios in samples collected from breastfeeding women from different regions of the West Bank of Palestine. Associations between sociodemographic characteristics with breast milk lead levels and M/B lead ratios were also investigated.

## Methods

### Study design

This study was conducted in a cross-sectional observational design in the period between October 2017 and April 2018. The study adhered to the Strengthening the Reporting of Observational Studies in Epidemiology (STROBE) Statement for reporting observational studies.

### The study participants and sample size calculation

The study participants were breastfeeding women who visited the primary healthcare in different regions of the West Bank of Palestine. The inclusion criteria were: 1) 18 years old or above, 2) healthy, 3) had term delivery, 4) breastfeeding for more than 15 days, 5) not taking chronic medications, and 6) willing to provide a written informed consent. Lactation period of more than 15 days was selected to avoid collecting colostrum because colostrum is rich in proteic and impoverished in fat contents compared to mature breast milk [7]. Mature breast milk samples were obtained to minimize variations in contents that could have an impact on the lead levels to be quantified. Before sample collection, women were asked to clean the breast are using alcohol wipes. The milk samples were collected by the women themselves by hand expression method [7]. In this study, foremilk samples were collected. The decision to obtain foremilk was based on the fact the first part of the milk obtained at the beginning of the breastfeeding session (foremilk) contains less fats than the milk obtained at the end of the breastfeeding session (hindmilk). To minimize variability in the contents and flow rate, the breast milk samples were collected in the period between 8:00 am to 11:00 am. The number of samples to be collected in this study was calculated at a 95% confidence interval (CI) using eq. (1) that is commonly used to estimate sample size in health sciences [17]:

1 $$ Sample\ size\ (n)=\frac{\ {Z}^2\ {\sigma}^2}{D^2} $$

The value of Z at 95% CI = 1.96. σ = standard deviation of lead level the biological matrix. The standard deviations of maternal blood lead level, breast milk lead level, and the M/B lead ratio were obtained from Ettinger et al. [14]. As the inductively coupled plasma-mass spectrometric (ICP-MS) method used in this study was highly sensitive, the sample error (D) was set at 1 [12]. As the values of Z and D in the equation are constant, the sample size needed for this study would depend on the value of σ. Assuming a σ value of 4.2, at least 68 samples would be needed for this study.

### Data collection

The design and objectives of the study were explained to the potential participants before their written informed consent was obtained. The participants were interviewed in privacy and a standard questionnaire was used to collect their sociodemographic characteristics. The questionnaire used in this study was modified from previous questionnaires used to collect basic sociodemographic characteristics of the participants [7, 12, 16]. As the participants in this study were Arab natives, the language of the questionnaire was Arabic. In this study, the questionnaire aimed to collect basic sociodemographic characteristics of the participants, therefore, the language used in the questionnaire was at a reading level suitable for 8th grade or lower educational level. The questionnaire was pilot tested for readability and comprehension among five women who had an educational level of 8th grade or lower. Based on the feedback obtained during the pilot testing, some items were rephrased for clarity. To ensure reliability of the questionnaire, the test-retest method was used in which 15 women who had an educational level of 8th grade or lower were asked to respond to the questionnaire twice. The time interval between the two rounds was between 30 min and 3 hours. Numeric answers of the women in both rounds were correlated using Pearson’s correlations as in previous studies [18]. The correlation coefficient (Pearson’s r) was 0.96 (95% CI 0.94–0.98) which indicated excellent reliability. The questionnaire collected characteristics like age, educational level, employment status, husband’s occupation, monthly household income, place of residence, number of children, and breastfeeding duration. The questionnaire also collected potential sources of lead exposure like distance of from a paint shop, industrial zone, and gas station, if the house paint was peeling/chipping, if the breastfeeding women worked in agriculture, used cosmetics, *eye kohl* (which is an eye cosmetic commonly worn by women in the Middle East, Africa, and the Indian Subcontinent), hair dye, and clay utensils. Breastfeeding women were also asked if they ever smoked or consumed alcohol. The characteristics collected in this study were similar to those collected in previous studies [7, 12]. The questionnaire is provided in Additional file 1.

About 5 mL of venous blood and 5 mL of breast milk were collected from each breastfeeding woman in heavy metal free polyethylene tubes that were incubated in 10% nitric acid (HNO_3_) for 24 h before use. The blood samples were withdrawn by practicing nurses who were licensed to perform phlebotomy. The samples collected were kept at 4 °C and shipped to the laboratory for storage at − 20 °C until the time of analysis.

### Analytical procedure

The tubes and containers that were used in this study were incubated overnight in 10% HNO_3_ to prevent adsorption of lead onto the surfaces. All chemicals used in this study were of analytical grade and obtained from Sigma-Aldrich (Darmstadt, Germany). Samples were analyzed using a validated ICP-MS method (Perkin Elmer Elan 9000) [14]. The method used was previously described in detail [14]. Samples analyzed in this study were vortexed for 10 s. Matrix/buffer mixtures of 0.15 mL were added to 0.15 mL concentrated HNO_3_. Mixtures were heated at 100 °C for 1 h. Mixtures were cooled before they were subsequently vortexed and diluted to a ratio of 1:10. The samples contained 0.25 mL of the acid-digested matrix, 0.75 mL MilliQ water (Merck Millipore Milli-Q™), and 1.5 mL of acid diluent. The acid diluent was 1% v/v concentrated HNO_3_. The internal standard was platinum (10 μg/L).

### Statistical analysis

Data collected in this study were entered into IBM SPSS for Windows v.21.0 (IBM, Arrmonk, NY, USA). The data were assessed for normality of distribution using the Kolmogorov-Smirnov test. As the data were not normally distributed, the first quartile (Q1), second quartile (Q2 = median), and third quartile (Q3) were used to describe the data. Results are presented as median (Q1, Q3). Categorical data were compared using Mann–Whitney *U* test. Breast milk lead levels were categorized into < 5 μg/L and ≥ 5 μg/L. Breast milk lead levels ≥5 μg/L were considered above the World Health Organization’s recommended levels [7, 13, 16]. Breast milk to blood lead ratios were categorized into < 0.25 and ≥ 0.25. Based on previous studies, this ratio indicated large M/B ratio [2, 16, 19]. Chi-square (χ^2^) or Fisher’s exact test was used to compare lead levels in these categories, as appropriate. Correlations between blood, breast milk, and M/B lead ratios were investigated using Spearman rank correlations (Spearman’s rho). Predictors of breast milk lead levels of ≥5 μg/L and M/B lead ratios of ≥25% were identified using multivariate logistic regression. The variables with *p* - values of < 0.3 in the χ^2^ or Fisher’s exact test were retained in the multivariate logistic regression model. In this study, a Backward Stepwise Likelihood Ratio method was used. Hosmer and Lemeshow Test and Omnibus Tests of Model Coefficients were used to assess goodness-of-fit. Odds ratios (OR) with their 95% CI were calculated using the multivariate logistic regression model. Statistical significance was considered when the *p* - value was ≤0.05.

### Ethics approval and consent to participate

The study was conducted in compliance with the Declaration of Helsinki and in accordance with the rules and regulations followed at An-Najah National University. This study received ethical approval from the Institutional Review Board (IRB) of An-Najah National University, Nablus (Protocol # NNU-IRB-10-14). Participants signed a written informed consent.

## Results

### Study participants and samples analyzed

**Fig. 1:**
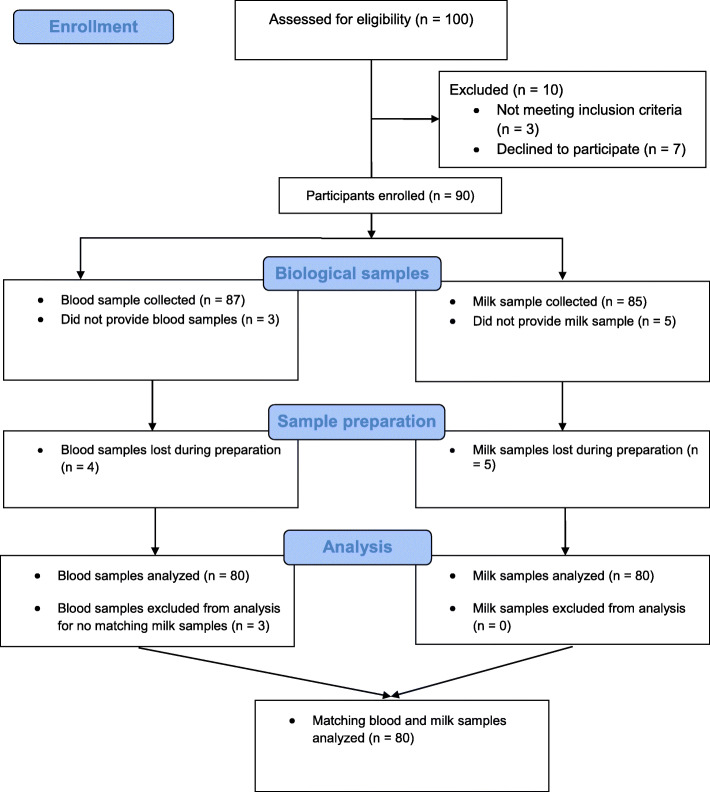
Flowchart of data collection and analysis

**Fig. 2:**
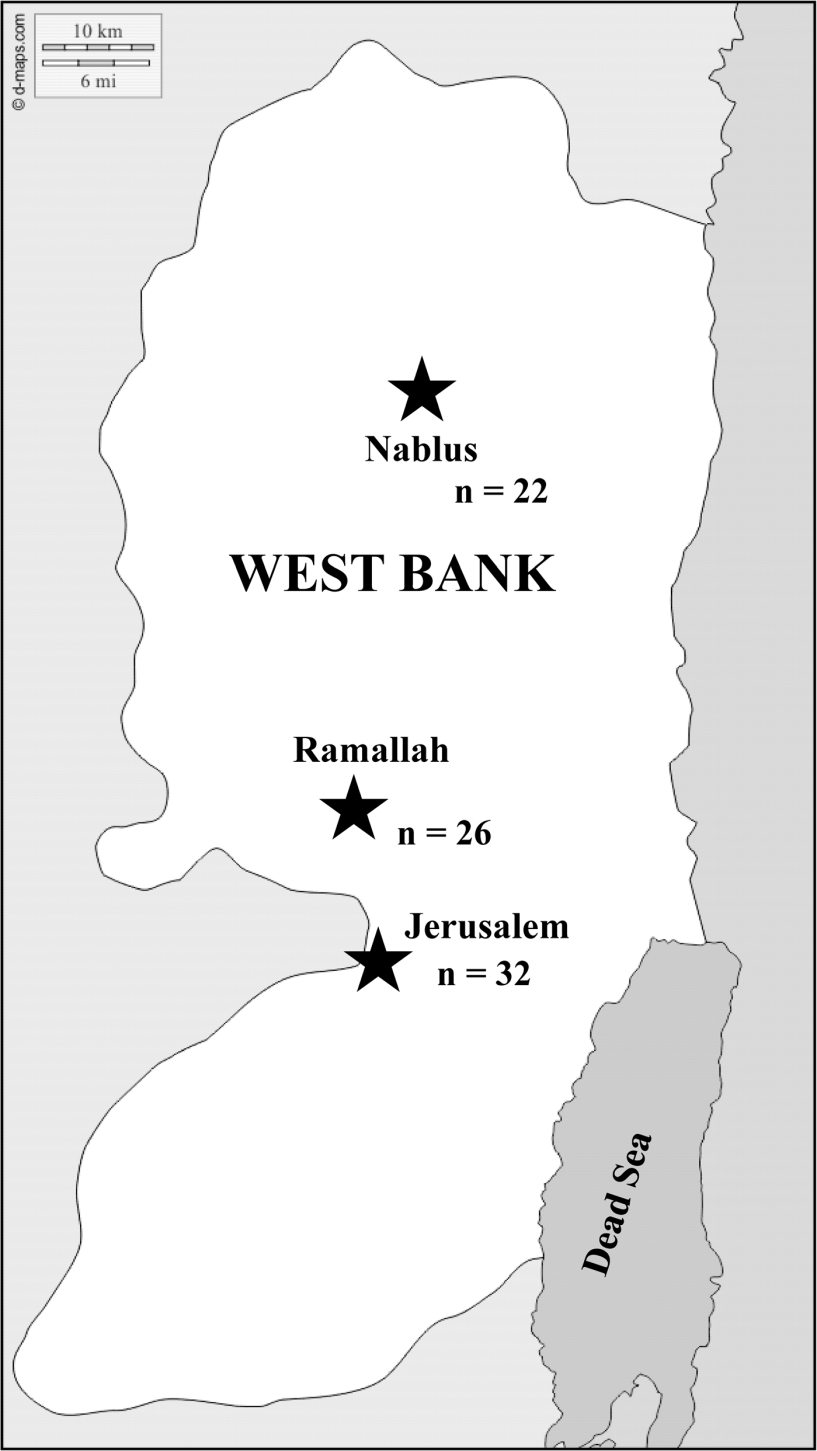
Regions from where participants were recruited

In this study, a total of 100 breastfeeding women were screened for eligibility. Of those, 10 were excluded and 90 breastfeeding women consented to provide blood and milk samples. Finally, matching blood and breast milk samples from 80 women were obtained and analyzed for their lead concentrations. Details of the participant enrollment process is shown in Fig. 1. The number of samples obtained from each region is shown in Fig. 2.

The median age of breastfeeding women was 27 (23, 30) years, the median number of children was 2 (1, 4), and the median breastfeeding duration was 7 (2, 10) months. All breastfeeding women declared that they did not consume alcohol and 1 (1.25%) was a smoker. The detailed sociodemographic variables of the breastfeeding women who participated in the study are shown in Table 1.

**Table 1:** Sociodemographic variables of the women who provided blood and breast milk samples (*n = 80*)

Variable	***n***	%
**Age (years)**		
< 25	35	43.8
≥ 25	45	56.3
**Number of children**		
≤ 2	43	53.8
> 2	37	46.3
**Breastfeeding duration (months)**		
< 6	34	42.5
≥ 6	46	57.5
**Place of residence**		
Rural areas	52	65.0
Urban areas	28	35.0
**Chipping house paint**		
No	54	67.5
Yes	26	32.5
**Distance from paints shop**		
Close (< 200 m)	15	18.8
Far (> 200 m)	65	81.3
**Distance from industrial area**		
Close (< 200 m)	22	27.5
Far (> 200 m)	58	72.5
**Distance from gas station**		
Close (< 200 m)	12	15.0
Far (> 200 m)	68	85.0
**Monthly household income (US$)**		
< 1000	64	80.0
≥ 1000	16	20.0
**Educational level**		
School	54	67.5
University	26	32.5
**Employment**		
Unemployed	70	87.5
Employed	10	12.5
**Husband’s occupation**		
White-collar job^a^	23	28.8
Blue-collar job^b^	57	71.3
**Worked in agriculture**		
No	25	31.3
Yes	55	68.8
**Use of cosmetics**		
No	46	57.5
Yes	34	42.5
**Use of kohl**^**c**^		
No	4	5.0
Yes	76	95.0
**Use of hair dye**		
No	38	47.5
Yes	42	52.5
**Use of clay utensils**		
No	76	95.0
Yes	4	5.0

^a^White-collar job was any job that does not involve physical labor, ^b^Blue-collar job was any job that involved manual/physical labor, ^c^*Eye kohl* is an eye cosmetic commonly worn by women in the Middle East, Africa, and the Indian Subcontinent

### Lead concentrations in blood, breast milk, and breast milk to blood (M/B) lead ratios

The median blood lead level was 1.00 μg/dL (0.65, 1.40), the median breast milk lead level was 0.40 μg/dL (0.20, 0.40), and the median M/B lead ratio was 0.33 (0.20, 0.50). One blood sample (1.3%) and 11 breast milk samples (13.8%) contained lead concentrations of ≥5 μg/dL and ≥ 5 μg/L, respectively. Blood lead concentrations correlated positively with breast milk lead concentrations (Spearman’s rho = 0.37, *p* - value = 0.001) and negatively with M/B lead ratios (Spearman’s rho = − 0.49, *p* - value < 0.001). Breast milk lead concentrations correlated positively with M/B lead ratios (Spearman’s rho = 0.57, *p* - value < 0.001).

Breast milk lead levels were significantly higher in breastfeeding women who lived in urban areas (*p* - value = 0.003) and those who lived close to industrial areas (*p* - value = 0.044) compared to those who lived in the rural areas and far from industrial areas. Breast milk to blood lead ratios were significantly higher in breastfeeding women who lived in urban areas (*p* - value = 0.026) and those who used *eye kohl* (*p* - value = 0.032) compared to those who lived in rural areas and did not use *eye kohl*. However, there was no evidence that blood lead levels were associated with any of the participants’ sociodemographic characteristics. Details of the lead concentrations blood, breast milk, and M/B lead ratios are shown in Table 2.

**Table 2:** Lead concentrations in blood, breast milk, and breast milk to blood (M/B) lead ratios

Variable	***n***	%	Blood lead level (μg/dL)		Milk lead level (μg/dL)		M/B ratio	
			Median (Q1, Q3)	***p***-value	Median (Q1, Q3)	***p***-value	Median (Q1, Q3)	***p***-value
**Age (years)**								
25	35	43.8	1.00 (0.80, 1.50)	0.56	0.40 (0.20, 0.40)	0.94	0.33 (0.20, 0.50)	0.88
≥ 25	45	56.3	1.00 (0.60, 1.40)		0.40 (0.20, 0.40)		0.33 (0.20, 0.43)	
**Number of children**								
≤ 2	43	53.8	1.00 (0.80, 1.40)	0.91	0.40 (0.20, 0.40)	0.96	0.33 (0.20, 0.50)	0.69
> 2	37	46.3	1.00 (0.60, 1.40)		0.40 (0.20, 0.40)		0.33 (0.20, 0.40)	
**Breastfeeding duration (months)**								
< 6	34	42.5	1.00 (0.60, 1.25)	0.07	0.40 (0.20, 0.40)	0.87	0.37 (0.24, 0.50)	0.05
≥ 6	46	57.5	1.20 (0.80, 1.40)		0.40 (0.20, 0.40)		0.33 (0.17, 0.40)	
**Place of residence**								
Rural areas	52	65.0	1.00 (0.70, 1.30)	0.45	0.20 (0.20, 0.40)	0.00	0.31 (0.19, 0.40)	0.03
Urban areas	28	35.0	1.10 (0.70, 1.60)		0.40 (0.20, 0.60)		0.40 (0.29, 0.50)	
**Chipping house paint**								
No	54	67.5	1.00 (0.80, 1.40)	0.77	0.20 (0.20, 0.40)	0.19	0.33 (0.20, 0.40)	0.11
Yes	26	32.5	1.00 (0.60, 1.40)		0.40 (0.20, 0.40)		0.37 (0.25, 0.57)	
**Distance from paints shop**								
Close (< 200 m)	15	18.8	1.20 (0.80, 1.40)	0.21	0.40 (0.20, 0.40)	0.46	0.33 (0.20, 0.43)	0.92
Far (> 200 m)	65	81.3	1.00 (0.60, 1.40)		0.20 (0.20, 0.40)		0.33 (0.20, 0.50)	
**Distance from industrial area**								
Close (< 200 m)	22	27.5	1.20 (0.80, 1.40)	0.14	0.40 (0.20, 0.40)	0.04	0.33 (0.20, 0.43)	0.94
Far (> 200 m)	58	72.5	1.00 (0.60, 1.40)		0.20 (0.20, 0.40)		0.33 (0.20, 0.50)	
**Distance from gas station**								
Close (< 200 m)	12	15.0	1.20 (0.90, 1.40)	0.17	0.40 (0.20, 0.40)	0.53	0.27 (0.17, 0.42)	0.34
Far (> 200 m)	68	85.0	1.00 (0.60, 1.40)		0.30 (0.20, 0.40)		0.33 (0.20, 0.50)	
**Monthly household income (US$)**								
< 1000	64	80.0	1.00 (0.70, 1.40)	0.83	0.20 (0.20, 0.40)	0.06	0.33 (0.20, 0.45)	0.17
≥ 1000	16	20.0	1.10 (0.70, 1.30)		0.40 (0.30, 0.40)		0.40 (0.29, 0.50)	
**Educational level**								
School	54	67.5	1.00 (0.60, 1.40)	0.95	0.40 (0.20, 0.40)	0.83	0.33 (0.20, 0.50)	0.68
University	26	32.5	1.00 (0.80, 1.20)		0.30 (0.20, 0.40)		0.33 (0.20, 0.50)	
**Employment**								
Unemployed	70	87.5	1.00 (0.80, 1.40)	0.12	0.40 (0.20, 0.40)	0.76	0.33 (0.20, 0.43)	0.10
Employed	10	12.5	8.00 (0.60, 1.20)		0.40 (0.20, 0.40)		0.45 (0.33, 0.50)	
**Husband’s occupation**								
White-collar job	23	28.8	1.00 (0.60, 1.30)	0.27	0.40 (0.20, 0.40)	0.42	0.40 (0.33, 0.50)	0.06
Blue-collar job	57	71.3	1.00 (0.80, 1.40)		0.40 (0.20, 0.40)		0.33 (0.20, 0.40)	
**Worked in agriculture**								
No	25	31.3	1.00 (0.70, 1.40)	0.62	0.40 (0.20, 0.40)	0.07	0.33 (0.25, 0.50)	0.05
Yes	55	68.8	1.00 (1.00, 1.40)		0.40 (0.20, 0.40)		0.29 (0.17, 0.40)	
**Use of cosmetics**								
No	46	57.5	1.00 (0.60, 1.40)	0.64	0.30 (0.20, 0.40)	0.63	0.33 (0.20, 0.50)	0.40
Yes	34	42.5	1.00 (0.80, 1.40)		0.40 (0.20, 0.40)		0.37 (0.20, 0.50)	
**Use of kohl**^**a**^								
No	4	5.0	1.30 (0.90, 2.30)	0.34	0.20 (0.20, 0.30)	0.23	0.16 (0.14, 0.25)	0.03
Yes	76	95.0	1.00 (0.70, 1.40)		0.40 (0.20, 0.40)		0.33 (0.20, 0.50)	
**Use of hair dye**								
No	38	47.5	1.00 (0.60, 1.40)	0.52	0.20 (0.20, 0.30)	0.33	0.33 (0.25, 0.40)	0.59
Yes	42	52.5	1.00 (0.80, 1.40)		0.40 (0.20, 0.40)		0.33 (0.20, 0.50)	
**Use of clay utensils**								
No	76	95.0	1.00 (0.60, 1.40)	0.37	0.40 (0.20, 0.40)	0.23	0.33 (0.20, 0.50)	0.08
Yes	4	5.0	1.20 (1.10, 1.30)		0.20 (0.20, 0.30)		0.19 (0.16, 0.27)	

^a^*Eye kohl* is an eye cosmetic commonly worn by women in the Middle East, Africa, and the Indian Subcontinent, Q1: 1st quartile, Q2: second quartile (median), Q3: 3rd quartile

### Association between sociodemographic variables, breast milk lead levels, and breast milk to blood (M/B) lead ratios

Chi-square/Fisher’s exact test showed that there was a significant association between breast milk lead level and urban residence. There was a significant association between M/B lead ratios with urban residence, working in agriculture, use of *eye kohl*, and use of clay utensils. Details of the associations are shown in Table 3.

**Table 3:** Association between sociodemographic variables, breast milk lead levels, and breast milk to blood lead ratios of the study participants (*n = 80*)

Variable	N	%	Breast milk lead concentration						Breast milk/venous blood lead ratio					
			< 5 μg/L		≥ 5 μg/L		χ^**2**^/Fisher’s exact test	***p***-value	< 25%		≥ 25%		χ^**2**^/Fisher’s exact test	***p***-value
			***n***	%	***n***	%			***n***	%	***n***	%		
**Age (years)**														
< 25	35	43.8	30	37.5	5	6.3	0.02	0.58	12	15.0	23	28.8	0.54	0.46
≥ 25	45	56.3	39	48.8	6	7.5			12	15.0	33	41.3		
**Number of children**														
≤ 2	43	53.8	36	45.0	7	8.8	0.5	0.53	14	17.5	29	36.3	0.29	0.59
> 2	37	46.3	33	41.3	4	5.0			10	12.5	27	33.8		
**Breastfeeding duration (months)**														
< 6	34	42.5	29	36.3	5	6.3	0.05	0.54	8	10.0	26	32.5	1.18	0.33
≥ 6	46	57.5	40	50.0	6	7.5			16	20.0	30	37.5		
**Residence**														
Rural areas	52	65.0	49	61.3	3	3.8	7.62	0.01	20	25.0	32	40.0	5.07	0.02
Urban areas	28	35.0	20	25.0	8	10.0			4	5.0	24	30.0		
**Chipping house paint**														
No	54	67.5	48	60.0	6	7.5	0.93	0.26	18	22.5	36	45.0	0.88	0.35
Yes	26	32.5	21	26.3	5	6.3			6	7.5	20	25.0		
**Distance from paints shop**														
Close (< 200 m)	15	18.8	14	17.5	1	1.3	0.77	0.46	3	3.8	12	15.0	0.88	0.35
Far (> 200 m)	65	81.3	55	68.8	10	12.5			21	26.3	44	55.0		
**Distance from industrial area**														
Close (< 200 m)	22	27.5	18	22.5	4	5.0	0.48	0.72	6	7.5	16	20.0	0.11	0.74
Far (> 200 m)	58	72.5	51	63.8	7	8.8			18	22.5	40	50.0		
**Distance from gas station**														
Close (< 200 m)	12	15.0	11	13.8	1	1.3	0.35	0.69	4	5.0	8	10.0	0.07	0.79
Far (> 200 m)	68	85.0	58	72.5	10	12.5			20	25.0	48	60.0		
**Monthly household income (US$)**														
< 1000	64	80.0	56	70.0	8	10.0	0.42	0.69	21	26.3	43	53.8	1.21	0.27
≥ 1000	16	20.0	13	16.3	3	3.8			3	3.8	13	16.3		
**Educational level**														
School	54	67.5	47	58.8	7	8.8	0.09	1.00	16	20.0	38	47.5	0.01	0.92
University	26	32.5	22	27.5	4	5.0			8	10.0	18	22.5		
**Employment**														
Unemployed	70	87.5	60	75.0	10	12.5	0.13	1.00	23	28.8	47	58.8	2.18	0.14
Employed	10	12.5	9	11.3	1	1.3			1	1.3	9	11.3		
**Husband’s occupation**														
White-collar job	23	28.8	18	22.5	5	6.3	1.72	0.28	4	5.0	19	23.8	2.44	0.12
Blue-collar job	57	71.3	51	63.8	6	7.5			20	25.0	37	46.3		
**Worked in agriculture**														
No	55	68.8	45	56.3	10	12.5	2.88	0.16	12	15.0	43	53.8	5.61	0.02
Yes	25	31.3	24	30.0	1	1.3			12	15.0	13	16.3		
**Use of cosmetics**														
No	34	42.5	29	36.3	5	6.3	0.05	1.00	9	11.3	25	31.3	0.35	0.55
Yes	46	57.5	40	50.0	6	7.5			15	18.8	31	38.8		
**Use of kohl**^**a**^														
No	4	5.0	4	5.0	0	0.0	0.66	0.64	3	3.8	1	1.3	4.06	0.04
Yes	76	95.0	65	81.3	11	13.8			21	26.3	55	68.8		
**Use of hair dye**														
No	38	47.5	32	40.0	6	7.5	0.25	0.75	15	18.8	23	28.8	3.09	0.08
Yes	42	52.5	37	46.3	5	6.3			9	11.3	33	41.3		
**Use of clay utensils**														
No	76	95.0	65	81.3	11	0.0	0.66	0.64	21	26.3	55	68.8	4.06	0.04
Yes	4	5.0	4	5.0	0	13.8			3	3.8	1	1.3		

^a^*Eye kohl* is an eye cosmetic commonly worn by women in the Middle East, Africa, and the Indian Subcontinent

### Predictors of higher breast milk lead levels and breast milk to blood (M/B) lead ratios

To control confounding variables and identify significant predictors of breast milk lead levels and M/B lead ratios of ≥25%, a multivariate logistic analysis was conducted. The multivariate logistic analysis showed that breast milk lead levels were more likely to be ≥5 μg/L in breastfeeding women who lived in urban areas (aOR 4.96; 95% CI 1.10, 22.38) compared to those who lived in rural areas. M/B lead ratios were more likely to be ≥25% in breastfeeding women who lived in urban areas (aOR 7.06; 95% CI 1.68, 29.77), used *eye kohl* (aOR 14.29; 95% CI 1.32, 155.06), and used hair dye (aOR 5.33; 95% CI 1.58, 18.00) compared to those who lived in rural areas, did not use *eye kohl*, and did not use hair dye. The details of the analysis are shown in Table 4.

**Table 4:** Predictors of higher breast milk lead levels and breast milk to blood (M/B) lead ratios

Variable	β	SE	Wald	***p***-value	OR	95% CI for OR	
						Lower	Upper
**Breast milk lead level**							
Urban residence	1.60	0.77	4.34	0.04	4.96	1.10	22.38
Worked in agriculture	0.97	1.15	0.70	0.40	2.63	0.27	25.11
Constant	−3.43	1.05	10.65	0.00	0.03		
**M/B lead ratios**							
Urban residence	1.95	0.73	7.09	0.01	7.06	1.68	29.77
Use of *eye kohl*^a^	2.66	1.22	4.78	0.03	14.29	1.32	155.06
Use of hair dye	1.67	0.62	7.26	0.01	5.33	1.58	18.00
Use of clay utensils	2.39	1.24	3.70	0.05	10.87	0.96	123.48
Constant	−5.23	1.83	8.13	0.00	0.01		

^a^*Eye kohl* is an eye cosmetic commonly worn by women in the Middle East, Africa, and the Indian Subcontinent. *CI* Confidence interval, *aOR* adjusted odds ratio to control for confounding variables, *SE* Standard error. A Backward Stepwise Likelihood Ratio method was used. The goodness of fit was good as indicated by a statistically non-significant Chi-square’s value of 0.58, *p*-value = 0.902 (Hosmer and Lemeshow Test) and a statistically significant model’s Chi-square’s value of 21.0, *p*-value < 0.001

## Discussion

This study reported blood lead levels, breast milk lead levels, and M/B lead ratios among Palestinian breastfeeding women. To the best of our knowledge, this study was the first to investigate M/B lead ratios among breastfeeding women in Palestine. In this study, the sampling method ensured representation of the entire population of breastfeeding women in the West Bank in terms of age groups, number of children, place of residence, income level, educational level, and employment status. As a middle-income country, a considerable percentage of employees in Palestine earned 1000 US$ or less per month. It is noteworthy mentioning that women represented about 16% of the labor force in Palestine and about 25% of the Palestinian women hold some university or college education.

In previous studies, lead levels were assessed in different biological matrices like venous blood, umbilical blood, plasma, serum, saliva, breast milk, and urine [7, 11–13]. However, conflicting findings were reported with regard to correlation between blood and breast milk lead concentrations [2, 14–16]. Findings of this study were contradictory to those reported by Nriagu et al. in which the researchers suggested that transfer of lead from plasma and bone to breast milk of breastfeeding women was low [20]. The authors proposed that the mammary gland could represent an efficient barrier that can limit transfer of lead to breast milk. On the other hand, findings of this study were consistent with those reporting correlations between blood and breast milk lead levels [16]. Additionally, results of this study supported findings of a previous study from Palestine in which transfer from plasma to milk appeared to be high at even low plasma lead levels [7]. Findings of this study also support the hypothesis that breastfeeding women living in low- and middle-income countries could be exposed to lead [7, 13, 20, 21]. It has been argued that fetuses could be subjected to lead exposure in utero as a result of maternal exposure [22, 23]. Infants fed lead-contaminated breast milk could also be an additional risk of exposure. According to the Centers for Disease Control and Prevention (CDC), healthcare providers should investigate sources of exposure in breastfeeding women having blood lead levels of 5 μg/dL or more [24]. It has been reported that maternal blood lead levels of 10 g/dL and more can increase the likelihood of spontaneous abortion, congenital malformations, and delays in the neurobehavioral development of offspring [22, 23].

Previous studies reported that lead levels in maternal blood and breast milk could vary significantly [2, 7, 13, 25] The variability could be explained by the level of exposure and the analytical method used [7, 13]. A systematic review of blood lead levels in African women of childbearing age reported mean blood lead levels of as high as 99 ± 123 μg/dL [25]. Another systematic review of breast milk lead level in Iran reported a median of about (23.1–68.1) μg/L [13]. Findings of this study showed that 13.8% of the breast milk samples contained lead levels above the World Health Organization’s recommended levels. This percentage was slightly lower than the one reported in a previous study in Palestine in which about 19% of the breast milk samples contained lead levels above the World Health Organization’s safe levels. In Iran, Vahidinia et al. reported that 94% of the breast milk samples contained lead levels above the World Health Organization’s recommended levels [13]. Findings of this study might indicate that the samples used were from occupationally unexposed breastfeeding women.

In this study, breast milk lead concentrations and M/B lead ratios were significantly higher in breastfeeding women living in urban areas and close to industrial areas. Findings of this study were consistent with those reported in a previous study in Palestine [7]. After controlling for confounding variables, M/B lead ratios of ≥25% were predicted by living in urban areas, using hair dyes, and using *eye kohl*. Compared to the rural areas, environmental pollution is significantly higher urban areas. Additionally, urban areas tend to have higher population densities, heavier traffic, and more industrial areas. Taken together, living closer to sources of pollution could expose breastfeeding women to higher lead levels [7]. Findings of this study showed that M/B lead ratios were higher in women who worked in agriculture, used *eye kohl*, and clay utensils. These findings were consistent with those previously reported on higher breast milk lead levels among Palestinian breastfeeding women [7]. Additionally, many previous studies have linked using clay and *eye kohl* with higher lead level [26, 27]. Analysis of different sources of *eye kohl* revealed higher proportions of lead [28]. In Palestine, cosmetics including *eye kohl* and hair dyes are loosely regulated and the markets are often flooded with many products of unknown quality and safety.

### Strengths and limitations of the study

Findings of this study could be interpreted after considering a number of strengths and limitations. First, the sample was collected from different areas of the West Bank of Palestine. This should have permitted diversifying the sociodemographic variables of the breastfeeding women included in this study. Second, the questionnaire used in this study collected the different sources of exposure to lead. This should have permitted comparing the findings of the current study with those reported in previous studies. Third, different statistical methods were used to facilitate interpreting the results obtained in this study. The use of advanced statistical tests should have permitted an in depth understanding of the associations between lead levels and various sociodemographic variables, especially, then the potentially confounding variables were controlled.

However, this study is not without limitations. First, a convenience sampling technique was used in this study to recruit potential participants from the breastfeeding women visiting the maternity care clinics in the West Bank of Palestine. Nonprobability sampling techniques could be inherently biased. The use of a probability sampling technique could have reduced the selection bias that could be associated with the sampling technique used in this study. Second, the information collected from the breastfeeding women were self-reported. The probability of certain social desirability responses could not be eliminated. Third, the sample size used in this study was small. Although, the sample size used in this study was calculated using standard deviation of lead levels reported in previous studies [14], this small sample size could also be considered a limitation of the regression model used. Fourth, consumption of vitamins and foods that could help reduce lead levels were not collected in this study. Previous studies have shown that vitamins and certain foods containing micronutrients could help detoxify lead.

## Conclusions

Findings of this study showed that breastfeeding women in Palestine were exposed to lead levels that could be detected and quantified in blood and breast milk. Higher M/B lead ratios were predicted by living in urban areas, using hair dye, and *eye kohl*. Findings of this study might advance knowledge of lead disposition in breastfeeding women, identify predictors of lower or higher M/B lead ratios, and assessment of risk of infant exposure. Additionally, findings of this study could be informative to decision makers in health authorities who might need to design measures to reduce exposure of breastfeeding women and their infants to lead. Decision makers in health authorities should address sources of exposure to lead in urban areas. Cosmetics containing lead should be assessed and regulated for their lead contents. Future studies are still needed to investigate how to design measures that could be effective in reducing lead exposure among breastfeeding women.

## Supplementary Information

**Additional file 1:.** The questionnaire.

## Data Availability

The datasets used and/or analyzed during the current study are available from the corresponding author on reasonable request.

## References

[1] Lessen R, Kavanagh K (2015). Position of the academy of nutrition and dietetics: promoting and supporting breastfeeding. J Acad Nutr Diet.

[2] Ettinger AS, Roy A, Amarasiriwardena CJ, Smith D, Lupoli N, Mercado-Garcia A (2014). Maternal blood, plasma, and breast milk lead: lactational transfer and contribution to infant exposure. Environ Health Perspect.

[3] Al-Saleh I, Moncari L, Jomaa A, Elkhatib R, Al-Rouqi R, Eltabache C (2020). Effects of early and recent mercury and lead exposure on the neurodevelopment of children with elevated mercury and/or developmental delays during lactation: a follow-up study. Int J Hyg Environ Health.

[4] Wani AL, Ara A, Usmani JA (2015). Lead toxicity: a review. Interdiscip Toxicol.

[5] World Health Organization (2010). Childhood lead poisoning.

[6] Agency for Toxic Substances and Disease Registry (2019). ATSDR’s substance priority list.

[7] Shawahna R, Zyoud A, Dwikat J, El-Helo M, Yacoub B, Hilal H (2016). Breast milk lead levels in 3 major regions of the West Bank of Palestine. J Hum Lact.

[8] Hanning RM, Sandhu R, MacMillan A, Moss L, Tsuji LJ, Nieboer E (2003). Impact on blood Pb levels of maternal and early infant feeding practices of first nation Cree in the Mushkegowuk territory of northern Ontario, Canada. J Environ Monit.

[9] Dala-Paula BM, Custódio FB, Knupp EAN, Palmieri HEL, Silva JBB, Glória MBA (2018). Cadmium, copper and lead levels in different cultivars of lettuce and soil from urban agriculture. Environ Pollut.

[10] Shen Z, Hou D, Zhang P, Wang Y, Zhang Y, Shi P, O'Connor D (2018). Lead-based paint in children's toys sold on China’s major online shopping platforms. Environ Pollut.

[11] Lemos VA, de Carvalho AL (2010). Determination of cadmium and lead in human biological samples by spectrometric techniques: a review. Environ Monit Assess.

[12] Shawahna R, Zyoud A, Naseef O, Muwafi K, Matar A. Salivary lead levels among workers in different industrial areas in the West Bank of Palestine: a cross-sectional study. Biol Trace Elem Res. 2021. 10.1007/s12011-020-02567-0. 10.1007/s12011-020-02567-0 33394307

[13] Vahidinia A, Samiee F, Faradmal J, Rahmani A, Taravati Javad M, Leili M (2019). Mercury, lead, cadmium, and barium levels in human breast milk and factors affecting their concentrations in Hamadan, Iran. Biol Trace Elem Res.

[14] Ettinger AS, Téllez-Rojo MM, Amarasiriwardena C, Bellinger D, Peterson K, Schwartz J, Hu H, Hernández-Avila M (2004). Effect of breast milk lead on infant blood lead levels at 1 month of age. Environ Health Perspect.

[15] Gulson BL, Jameson CW, Mahaffey KR, Mizon KJ, Patison N, Law AJ, Korsch MJ, Salter MA (1998). Relationships of lead in breast milk to lead in blood, urine, and diet of the infant and mother. Environ Health Perspect.

[16] Koyashiki GAK, Paoliello MMB, Tchounwou PB (2010). Lead levels in human milk and children's health risk: a systematic review. Rev Environ Health.

[17] Daniel WW, Cross CL, Daniel WW, Cross CL (2018). Determination of sample size for estimating means. Biostatistics: a foundation for analysis in the health sciences.

[18] Shawahna R, Samaro S, Ahmad Z (2021). Knowledge, attitude, and practice of patients with type 2 diabetes mellitus with regard to their disease: a cross-sectional study among Palestinians of the West Bank. BMC Public Health.

[19] Keller CA, Doherty RA (1980). Lead and calcium distributions in blood, plasma and milk of the lactating mouse. J Lab Clin Med.

[20] Nriagu J, Burt B, Linder A, Ismail A, Sohn W (2006). Lead levels in blood and saliva in a low-income population of Detroit, Michigan. Int J Hyg Environ Health.

[21] Murtaza G, Tariq M, Shawahna R, Akash MS, Rehman K, Hashmi MZ (2021). Mitochondrial dysfunction in metabolic disorders. Endocrine disrupting chemicals-induced metabolic disorders and treatment strategies.

[22] Hu H, Téllez-Rojo MM, Bellinger D, Smith D, Ettinger AS, Lamadrid-Figueroa H, Schwartz J, Schnaas L, Mercado-García A, Hernández-Avila M (2006). Fetal lead exposure at each stage of pregnancy as a predictor of infant mental development. Environ Health Perspect.

[23] Liu J, Gao D, Chen Y, Jing J, Hu Q, Chen Y (2014). Lead exposure at each stage of pregnancy and neurobehavioral development of neonates. Neurotoxicology.

[24] Centers for Disease Control and Prevention (2019). Lead.

[25] Bede-Ojimadu O, Amadi CN, Orisakwe OE. Blood lead levels in women of child-bearing age in Sub-Saharan Africa: a systematic review. Front Public Health. 2018;6(367). 10.3389/fpubh.2018.00367. 10.3389/fpubh.2018.00367 PMC6305709 30619808

[26] Keosaian J, Venkatesh T, D’Amico S, Gardiner P, Saper R (2019). Blood lead levels of children using traditional Indian medicine and cosmetics: a feasibility study. Glob Adv Health Med.

[27] Nakhaee S, Amirabadizadeh A, Zarban A, Nasirizade M, Salmani Mood M, Ataei H, Shariatmadari MR, Brent J, Mehrpour O (2019). The reference value of blood lead level among the general adult population of eastern Iran. J Environ Sci Health A Tox Hazard Subst Environ Eng.

[28] Filella M, Martignier A, Turner A (2020). Kohl containing lead (and other toxic elements) is widely available in Europe. Environ Res.

