# Supplementary material for: Breast milk to blood lead ratios among women from the West Bank of Palestine: a cross-sectional study of associated factors
Source: Int Breastfeed J. 2021 Aug 23;16:61. doi: 10.1186/s13006-021-00410-3 (PMC8381486; doi:10.1186/s13006-021-00410-3)
Supplement: Supplementary file 1 — Additional file 1:. The questionnaire. [file 13006_2021_410_MOESM1_ESM.docx]

**Supplementary files for the manuscript:**

**Breast milk to blood lead ratios among women from the West Bank of Palestine: a cross-sectional study of associated factors**

Ramzi Shawahna^1,2*^

^1^Department of Physiology, Pharmacology and Toxicology, Faculty of Medicine and Health Sciences, An-Najah National University, Nablus, Palestine

^2^An-Najah BioSciences Unit, Centre for Poisons Control, Chemical and Biological Analyses, An-Najah National University, Nablus, Palestine

**^*^Correspondence:**

Ramzi Shawahna, PhD, Department of Physiology, Pharmacology and Toxicology, Faculty of Medicine & Health Sciences, New Campus, Building: 19, Office: 1340, An-Najah National University, P.O. Box 7, Nablus, Palestine

Phone: + (970) 923 45113 ext 2772

Phone: + (970) 92349739

Email: [ramzi_shawahna@hotmail.com](mailto:ramzi_shawahna@hotmail.com)

**Supplementary File 1**

**The Questionnaire**

**Blood and breast milk lead levels among breastfeeding women from the West Bank**

1. Age in years: ……………………
2. Number of children: ……………….
3. Breastfeeding duration in months: …………….
4. Place of residence: □ Urban residence □ Rural residence
5. Do you smoke? □ No □ Yes, if yes, please provide the number of cigarettes per day: ………..
6. Do you consume alcohol? □ No □ Yes, if yes, please provide the number of liters per month: …
7. Living in a house with chipping paint: □ No □ Yes
8. Distance from your home to the nearest paints shop: □ < 200 m □ ≥ 200 m
9. Distance from your home to the nearest industrial area: □ < 200 m □ ≥ 200 m
10. Distance from your home to the nearest gas station: □ < 200 m □ ≥ 200 m
11. The monthly household income (Jordanian Dinner): □ < 750 Dinars □ ≥ 750 Dinars
12. Your highest educational level: □ School □ University
13. Are you employed: □ No □ Yes, if yes please provide your profession: ……………..
14. Is your husband employed: □ No □ Yes, if yes please provide his profession: ……………..
15. Did you work in agriculture? □ No □ Yes, if yes please provide for how many years: ………..
16. Do you use cosmetics quite often? □ No □ Yes
17. Do you use eye kohl quite often? □ No □ Yes
18. Do you use hair dyes quite often? □ No □ Yes
19. Do you use clay utensils quite often? □ No □ Yes
